# Hypomethylating agents plus venetoclax for high-risk MDS and CMML as bridge therapy to transplant: a GESMD study

**DOI:** 10.1186/s40164-025-00652-5

**Published:** 2025-04-26

**Authors:** Ines Zugasti, Monica Lopez-Guerra, Sandra Castaño-Díez, Daniel Esteban, Alejandro Avendaño, Helena Pomares, Ana Perez, Sara García-Ávila, Irene Padilla Conejo, Cristina de la Fuente Montes, Alexandra Martínez-Roca, Beatriz Merchán, Carlos Jiménez-Vicente, Francesca Guijarro, Jose Ramón Álamo, Albert Cortes-Bullich, Victor Torrecillas, Lucia Mont, Esther Carcelero, Gisela Riu, Lurdes Zamora, Joan Bargay, Ana Triguero, Maria Suarez-Lledó, Maria Queralt Salas, Felix López-Cadenas, Fernando Ramos, Blanca Xicoy, David Valcárcel, Montserrat Arnan, Carmen Martínez, Montserrat Rovira, Francesc Fernández-Avilés, Maria Díez-Campelo, Jordi Esteve, Marina Díaz-Beyá

**Affiliations:** 1 Hospital Clínic Barcelona, Barcelona, Spain; 2 Hospital Universitario de Salamanca, Salamanca, Spain; 3 Institut Català d’Oncologia, Hospital Duran I Reynals, L’Hospitalet de Llobregat, Barcelona, Spain; 4 Hospital Universitario Vall d´Hebrón, Barcelona, Spain; 5 Hospital del Mar, Barcelona, Spain; 6 Hospital Universitario de León, León, Spain; 7 Institut Català d’Oncologia (ICO), Hospital Germans Trias I Pujol, Badalona, Spain; 8 Universitat de Barcelona, Barcelona, Spain; 9 Hospital Son Llátzer, Palma, Spain; 10 Grupo Español de Síndromes Mielodisplásicos (GESMD), Madrid, Spain; 11 Fundació de Recerca Clínic Barcelona-Institut d’Investigacions Biomèdiques August Pi I Sunyer (FRCB-IDIBAPS), Barcelona, Spain; 12 Josep Carreras Leukemia Research Institute, Barcelona, Spain; 13 Centro de Investigación Biomédica en Red de Cáncer (CIBERONC), Madrid, Spain; 14 Universitat Autònoma de Barcelona, Barcelona, Spain

**Keywords:** MDS, CMML, MDS/MPN, Allo-SCT, Bridge therapy, Cytoreductive therapy, HMA/VEN, MRD, Molecular follow-up

## Abstract

**Background:**

High-risk myelodysplastic syndromes (HR-MDS) and chronic myelomonocytic leukemia (CMML) remain therapeutic challenges with suboptimal outcomes. The only potentially curative treatment is allogeneic stem cell transplantation (allo-SCT). The most frequent pre-allo-SCT treatment is monotherapy with hypomethylating agents (HMA), but approximately 40% of patients cannot proceed to allo-SCT, mainly due to disease progression. Recent evidence suggests that combining HMA with venetoclax (HMA/VEN) could increase HMA efficacy in HR-MDS but it remains unclear if this combination could bridge more patients to allo-SCT.

**Methods:**

We retrospectively evaluated HMA/VEN as a bridge to allo-SCT in 30 patients with HR-MDS or CMML eligible for transplant. Eighteen patients were treatment-naïve and 12 were refractory or relapsed (R/R).

**Results:**

As defined by the IWG 2023 criteria, the overall response rate (ORR) was 90% and the composite complete response rate was 77%. For the R/R patients, ORR was 83%. The allo-SCT rate was 83%, and the allo-SCT rate of those patients treated exclusively with HMA/VEN without further bridge therapies was 76%. One- and two-year post-allo-SCT survival was 75% and two-year cumulative incidence of relapse was 30.5%. Follow-up of measurable residual disease identified some molecular relapses that were controlled with preemptive treatment.

**Conclusions:**

Our findings indicate that HMA/VEN combination therapy shows promise as a bridging strategy to allo-SCT in HR-MDS and CMML.

**Supplementary Information:**

The online version contains supplementary material available at 10.1186/s40164-025-00652-5.

## Background

High-risk myelodysplastic syndromes (HR-MDS) and myelodysplastic syndromes/myeloproliferative neoplasms (MDS/MPN), such as chronic myelomonocytic leukemia (CMML) have a suboptimal prognosis, with an overall survival (OS) of less than 20 months [1–3]. The only potentially curative treatment at this time is allogeneic stem cell transplantation (allo-SCT), which has been shown to confer longer OS [4, 5]. Several challenges are associated with allo-SCT, including a significant percentage of patients who, despite the intention of undergoing allo-SCT, do not finally reach it [4] and a notably high rate of post-allo-SCT relapse [4, 6, 7]. Several studies suggest that a low pre-allo-SCT tumor burden is associated with longer post-transplant survival [8], indicating a potential benefit for pre-allo-SCT cytoreductive treatment. AML-like chemotherapy based on 3 + 7 scheme can be applied for HR-MDS, but generally present lower CR rates, shorter CR duration and tend to be associated with higher toxicity including more prolonged periods of aplasia than observed in AML [9].

The most frequent pre-allo-SCT treatment in patients with HR-MDS or CMML is monotherapy with hypomethylating agents (HMA), such as azacytidine (AZA) and decitabine (DEC) [10–13]. However, fewer than 20% of patients attain a complete response (CR) and fewer than 50% achieve any response [14]. Moreover, approximately 43% of patients die or progress before being able to undergo allo-SCT [4, 15], indicating a clear need for more effective bridging strategies. Therefore, there is controversy regarding whether pre-allo-SCT cytoreductive therapy is necessary and which scheme is the most efficacious. An ongoing phase II randomized clinical trial (NCT01812252) is comparing induction chemotherapy vs HMA as pre-allo-SCT therapy, but to the best of our knowledge, no other prospective clinical trials are addressing this issue [16].

The B-cell lymphoma 2 (BCL2) protein family plays a significant role in the intrinsic mitochondrial apoptosis pathway [17]. Most tumor stem cells in acute myeloid leukemia (AML), MDS, and CMML have high levels of BCL2 and are dependent on BCL2 for survival [18]. Venetoclax (VEN) is a BCL2 inhibitor that was explored in combination with AZA as a first-line treatment for elderly AML patients in a phase III randomized trial; OS was longer in patients receiving the combination therapy than in those receiving AZA alone [19]. HMA/VEN is now the standard first-line treatment in unfit AML patients.

HMA/VEN is now being investigated in MDS [20–25], where it has achieved a high overall response rate (ORR) of 70% and a median early response of 1–2 months. Refractory or relapsed (R/R) MDS patients previously treated with HMA have a dismal prognosis, with OS of less than 6 months [26]. Recently, however, R/R MDS patients treated with HMA/VEN achieved an ORR of 40–50% [21, 27, 28]. HMA/VEN has also achieved a high ORR in CMML patients, although there was also a high rate of early relapse in these patients [29–31]. These findings suggest that HMA/VEN could be an effective bridging strategy to allo-SCT for HR-MDS and CMML patients. Recently, a retrospective study suggested promising activity in 13 HR-MDS patients who received HMA/VEN and proceeded to allo-SCT [32]. Several small preliminary studies have also reported promising outcomes with pre-allo-SCT HMA/VEN [27, 28, 30, 33–35]. Additionally, an ongoing clinical trial is exploring the effectiveness and toxicity of this combination in MDS (VERONA NCT04401748). The VERONA study is not focused exclusively on patients who are candidates for allo-SCT, although the results will certainly shed some light on the impact of HMA/VEN on allo-SCT. However, real-world studies are still essential to determine whether combined therapy with HMA/VEN will yield a higher percentage of patients who are able to undergo allo-SCT.

To the best of our knowledge, no study has specifically evaluated the transplant rate of eligible patients who received HMA/VEN as a bridge to allo-SCT. We have performed a retrospective study of patients with HR-MDS or MDS/MPN who were eligible for allo-SCT and were treated with HMA/VEN as a bridging strategy. In this patient cohort, we have evaluated tolerability, response rate, allo-SCT transplant rate, OS, and post-allo-SCT outcomes including measurable residual disease (MRD) during post allo-SCT follow-up.

## Methods

### Study design

We included 30 HR-MDS or MDS/MPN patients who were eligible for allo-SCT and who were treated with off-label HMA/VEN as a bridge to allo-SCT. Both treatment-naïve and R/R patients were eligible for inclusion. This was a retrospective multicenter study of patients from eight hospitals that are part of the Spanish Group of Myelodysplastic Syndromes (GESMD – Grupo Español de Síndromes Mielodisplásicos).

Responses in MDS were evaluated according to the International Working Group (IWG) 2023 criteria [36]. ORR included complete remission (CR), partial remission (PR), CR with limited count recovery (CR_L_), CR with partial hematologic recovery (CRh), and hematological improvement (HI). The composite response rate (CRc) included CR, CR_L_ and CRh. Responses in CMML patients were evaluated according to the Savona criteria [37]. The IWG 2006 [38] and the European LeukemiaNet (ELN) 2022 [39] classifications were considered when comparing response rates. Data were collected retrospectively and updated in March 2024. Baseline characteristics were considered as those present at the moment of HMA/VEN initiation. All subjects gave their informed consent for inclusion in the study. The study was conducted in accordance with the Declaration of Helsinki, and the protocol was approved by the Institutional Review Board of Hospital Clínic Barcelona (Barcelona, Spain) (project identification code HCB/2023/0970).

### Cytogenetics and molecular assessment

Cytogenetics were analyzed in metaphase cells with G-banding using standard techniques. Karyotypes were reported according to the International System for Human Cytogenetic Nomenclature [40]. Molecular screening of FLT3-ITD, FLT3-TKD, and NPM1 mutations was performed by conventional PCR-based. FLT3-ITD allelic ratio was calculated using PCR DNA fragment analysis [41]. Targeted next-generation sequencing (NGS) was performed on genomic DNA with Ion AmpliseqTM AML Research Panel, the OncomineTM Myeloid Research Assay (ThermoFisher Scientific, Waltham, MA, USA), In-home Myeloid Pannel (Sophia Genetics), and Haematology OncoKitDx (Imegen®). Sequencing data were analyzed using Ion Reporter software (ThermoFisher Scientific) and MiSeqDx (Illumina) with a sensitivity of 2%. Follow-up with ddPCR was also measured after allo-SCT in 9 patients. This molecular monitoring of mutations identified at diagnosis assessed by droplet digital PCR (ddPCR) was made using specific assays (Bio‐Rad, Hercules, CA, USA). Briefly, 50 to 100 ng of genomic DNA samples were tested in duplicate on the Bio-Rad QX 200 QX200 Droplet Digital PCR platform. Data were then analyzed with QuantaSoft Software 1.0 (Bio‐Rad). For each particular case, the selection of MRD markers for ddPCR was done taking into account the ELN MRD Working Party recommendations [41], as well as the variant allele frequencies of the mutations detected at diagnosis and the feasibility of designing the ddPCR assay. Mutations assessed and its corresponding detection limit were as follows: ZRSR2 c.195_198del, detection limit 0,043; RUNX1 c.313_314insCG, detection limit 0,036; EZH2 R690H, 0,029; IDH2 R140Q, 0,035; SRSF2 P95H: detection limit 0,04; U2AF1 Q157P: detection limit 0,005; U2AF1 R156H: detection limit 0,005; JAK2 V617F: detection limit 0,03; BCOR C.413_414DUP: detection limit 0,03.

### Allo-SCT criteria

Eligibility criteria for allo-SCT, donor selection, conditioning regimen, and GVHD prophylaxis adhered to the standard practices of each of the eight participant institutions [42]. We included all eligible HR-MDS or HR- MDS/MPN treated with HMA/VEN with the intention of proceeding to allo-SCT. The intensity of the conditioning regimen was uniformly tailored to chronological age and comorbidities. No patient has received prophylactic post allo-SCT treatment. Grading of acute and chronic GVHD (aGVHD and cGVHD) followed established criteria [43, 44].

### Statistical analyses

OS was defined as the time between starting HMA/VEN and death from any cause. Post-allo-SCT OS was defined as the time between the time of allo-SCT until the last follow-up or death from any cause. Survival was calculated according to the Kaplan–Meier method and compared with the log-rank test. The cumulative incidence of relapse (CIR) was calculated with the Fine and Gray competing risk analysis with death without relapse as a competitive event. Non-relapse mortality was defined as death without recurrent or progressive disease after allo-SCT.

GVHD-free/relapse-free survival (GRFS) was defined as the duration from transplant until death, relapse, development of grade III-IV acute GVHD, or development of moderate/severe chronic GVHD requiring systemic treatment. Patients with none of these events at the time of the final follow-up were censored and GRFS was calculated using the Kaplan–Meier method. Fisher’s exact test was used to compare response rates between different groups. Statistical analyses were performed with R version 4.3.0 (RStudio, PBC, Boston, MA). All p-values were 2-sided, and significance was set at p ≤ 0.05.

## Results

### Patients and treatment

**Fig. 1:**
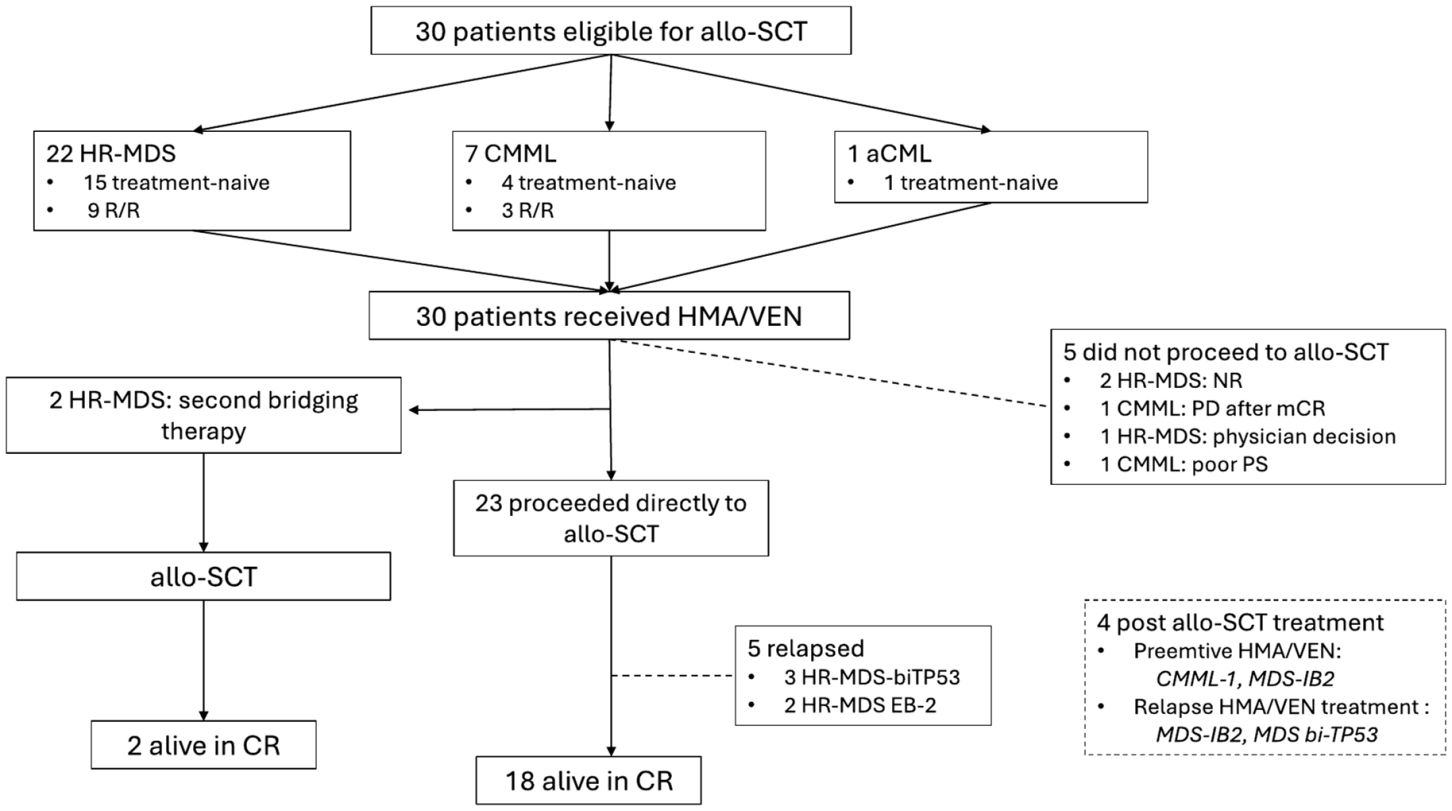
Flow chart of patients included in the study. *Allo-SCT* allogenic stem-cell transplant, *MDS* myelodysplastic syndrome, *CMML* chronic myelomonocytic leukemia, *aCML* atypical chronic myeloid leukemia, *NR* no response, *PD* progressive disease, *mCR* marrow complete response (by Savona criteria)

Figure 1 shows the flow chart of the patients included in the study. A total of 30 patients were included: 22 with HR-MDS and eight with MDS/MPN. Of the eight MDS/MPN patients, seven had CMML and one had atypical chronic myeloid leukemia (aCML). The median age at the time of starting HMA/VEN was 61.5 years (range, 41–74). Eighteen patients were treatment-naïve and 12 were R/R.

**Fig. 2:**
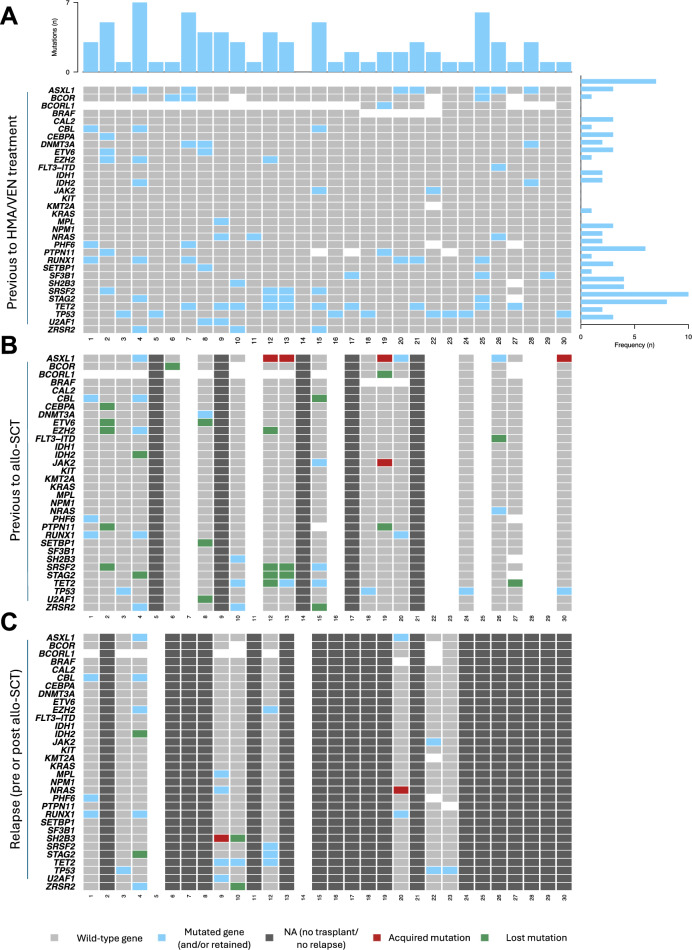
Oncoplot showing the mutational landscape of high-risk MDS/CMML patients **A** at baseline, **B** pre-allo-SCT, and **C** at relapse. Blue squares indicate mutations; grey squares indicate wild-type; white squares indicate untested; red squares indicate acquired mutations; green squares indicate lost mutations. The frequency (%) of each gene mutation can be seen on the right side of the plot. The number of mutations (n) is shown at the top

Of the 22 HR-MDS patients, nine were classified as very high risk, 11 as high risk and two as moderate-high risk according to the Molecular International Prognostic Scoring System (IPSS-M). Of the seven CMML patients, three were classified as high risk and four as intermediate-2 risk according to the CPSS-Mol classification. TP53 mutations were present in six patients (20%), all of whom had a complex karyotype. Baseline patient characteristics are shown in Table 1 and the baseline mutational profile in Fig. 2A*.*

**Table 1:** Baseline patient characteristics at the moment of starting HMA/VEN treatment

Characteristics	n = 30
Age, years, median (range)	61.5 (41–74)
Sex (male), n (%)	25 (83)
Treatment-related, n (%)	3 (10)
First line, n (%)	18 (60)
*Refractory/relapse, n (%)*	*12 (40)*
HMA	9
Lenalidomide	1
Idarubicin + Cytarabine	1
IDA-FLAG + allo-SCT	1
Leukocytes, × 10^9/L, median (range)	2.685 (0.9–43.7)
Neutrophils, × 10^9/L, median (range)	1.165 (0.14–7.9)
Monocytes, × 10^9/L, median (range)	0.108 (0–4.19)
Platelets, × 10^9/L, median (range)	69 (6–298)
Hemoglobin, g/L, median (range)	94 (60–136)
% Blasts in bone marrow, median (range)	10 (4–19)
*Cytogenetic risk in MDS, n (%)*	*n* = *20*
Good	7 (35)
Intermediate	2 (10)
Poor	8 (40)
Very poor	3 (15)
*Cytogenetic risk in CMML, n (%)*	*n* = *7*
Low	5 (71)
High	2 (29)
*2022 WHO Classification, n (%)*	*n* = *30*
MDS-IB1	4 (13)
MDS-IB2	11 (37)
MDS-bi*TP53*	6 (20)
MDS-*SF3B1*	1 (3)
CMML-1	3 (10)
CMML-2	4 (13)
MDS/MPN with neutrophilia	1(3)
*2022 ICC Classification, n (%)*	*n* = *30*
MDS-EB	4 (13)
MDS-NOS with multilineage dysplasia	1(3)
MDS-*TP53*mut	5 (17)
MDS-*SF3B1*	1 (3)
MDS/AML with myelodysplasia related mutations	8 (40)
MDS/AML with myelodysplasia related cytogenetic alterations	1(3)
MDS/AML NOS	2 (15)
CMML-1	3 (10)
CMML-2	4 (13)
aCML	1(3)
*CMML FAB Classification, n (%)*	*n* = *7*
Myelodysplastic	3 (43)
Myeloproliferative	4 (57)
*IPSS-Mol, n (%)*	*n* = *22*
Moderate-high	2 (9)
High	11 (50)
Very High	9 (41)
*CPSS-Mol, n (%)*	*n* = *7*
Intermediate-2	4 (57)
High	3 (43)

Previous treatment in the R/R patients was HMA as monotherapy in nine, lenalidomide in one, and chemotherapy in two, one of whom underwent allo-SCT after chemotherapy.

HMA consisted of AZA in 25 patients (83.4%) and DEC in five (16.6%). Duration of VEN treatment was 14 days in 21 patients, 21 days in one patient, 23 days in one patient, and 28 days in seven patients.

Only six patients (20%) received in-patient ramp-up. The remaining 24 patients were treated as outpatients, 18 of whom were included in an at-home care program for at least the first cycle of treatment (Table 2). Median number of cycles was 2 (range 1–9).

**Table 2:** Treatment details and toxicity profile in 30 patients receiving HMA/VEN

HMA/VEN TREATMENT (n = 30)	
*Type of hypomethylating agent, n (%)*	*n* = *30*
Azacytidine (75 mg/m2 sc, 7 days)	25 (83%)
Decitabine (20 mg/m2 i/v, 5 days)	5 (97%)
*Duration of venetoclax, first cycle (n, %)*	*n* = *30*
14 days	21 (70%)
21 days	1 (3%)
23 days	1 (3%)
28 days	7 (24%)
*Treatment modality, n (%)*	*n* = *29*
Outpatient	24 (80)
At-home care	18 (23)
Inpatient ramp-up	6 (20)
*Number of VEN/HMA cycles received, n (%)*	*n* = *30*
*1*	5 (17%)
*2*	11 (37%)
*3*	8 (27%)
*4*	4 (13%)
*5*	1 (3%)
*9*	1(3%)
*Safety Characteristics*	*n* = *30*
Use of G-CSF, n (%) (n = 30)	10 (33)
Use of antifungal prophylaxis, n (%)	14 (47)
Grade 3/4 neutropenia, n (%)	23 (85)
Grade 3/4 thrombocytopenia n (%)	19 (70)
Grade 3/4 anemia n (%)	17(63)
Febrile neutropenia, n (%)	5 (17)
Fungal infection, n (%)	0
Admission, n (%)	5 (17)
Delay of the next cycle n (%)	15 (50)
Days of delay of the next cycle, median (range)	19 (4–60)
30-days mortality	0
60-days mortality	1 (4%)

### Safety and tolerability

Table 2 and Supplementary Table 2 provides details on safety and tolerability. G-CSF was administered in ten patients (33%) and antifungal prophylaxis in 14 patients (47%). Grade 3/4 thrombocytopenia or neutropenia was reported in 19 (70%) and in 23 (85%) patients, respectively. Neutropenia occurred during the first cycle in all 23 patients, in the second cycle in 11 patients, and in the third cycle in nine patients. Five patients (17%) experienced febrile neutropenia requiring hospital admission – all during the first cycle. Of them, four were patients receiving HMA/VEN in a R/R status.

Fifteen patients (50%) required treatment delay. Median duration of treatment delay was 19 days (range, 4–60). One patient suffered a 60-day delay before the third cycle due to a non-hematological issue (lung nodule study), and another patient experienced a 50-day delay before the second cycle due to an oligosymptomatic COVID-19 infection with recovered counts that required three weeks to negativize the viral load. In the remaining 13 patients, the delay was 4–25 days due to recovering counts. Dose reduction of VEN was necessary in five patients due to grade 3/4 thrombocytopenia or neutropenia as detailed in Supplementary Table 2.

Thirty-day mortality was 0%, and one patient died within the first 60 days due to disease progression (4%).

### Response

**Fig. 3:**
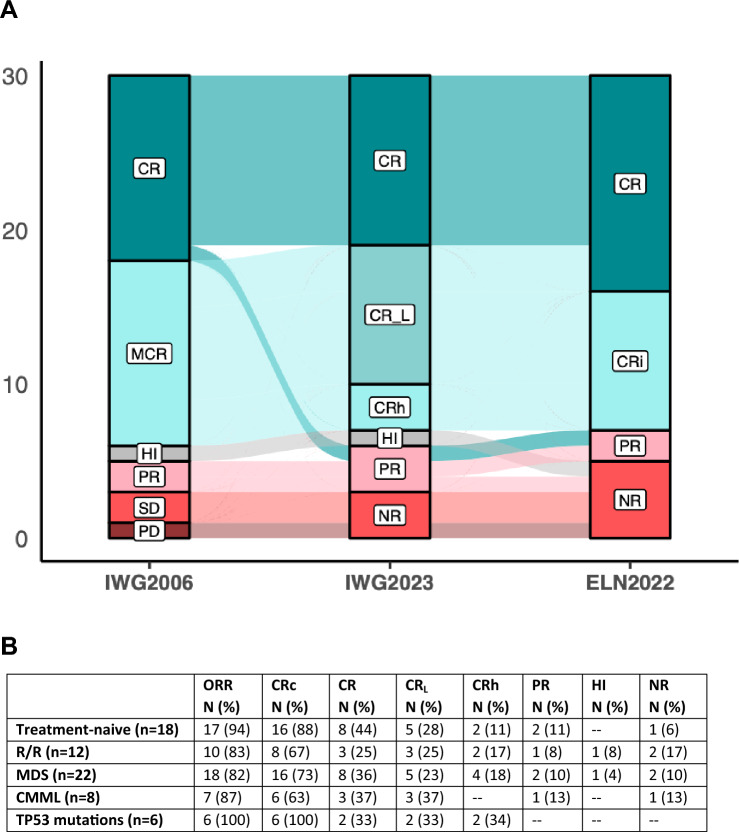
**A** Alluvial plot of the best responses achieved according to the International Working Group (IWG) 2006, IWG 2023, and European Leukemia Net (ELN) 2022 criteria. **B** Summary of responses for treatment-naïve patients, R/R patients, HR-MDS patients, CMML patients, and TP53-mutated patients. *CR* complete response, *CR*_*L*_ complete response with limited count recovery, *CRh* complete response with hematologic recovery, *CRi* complete response with incomplete recovery, *mCR* marrow complete response, *HI* hematologic improvement, *PR* partial response, *NR* no response, *PD* progressive disease, *ORR* overall response rate, *CRc* composite complete response rate

According to IWG 2023 criteria, ORR was 90% (27/30) and CRc was 77% (23/30). There were 11 CR (37%), eight CR_L_ (27%), four CRh (13%), three PR (10%), one HI (3%), and three no response (NR, 10%). ELN 2022 response criteria identified a higher rate of CR (46% [14/30]) (Fig. 3).

**Fig. 4:**
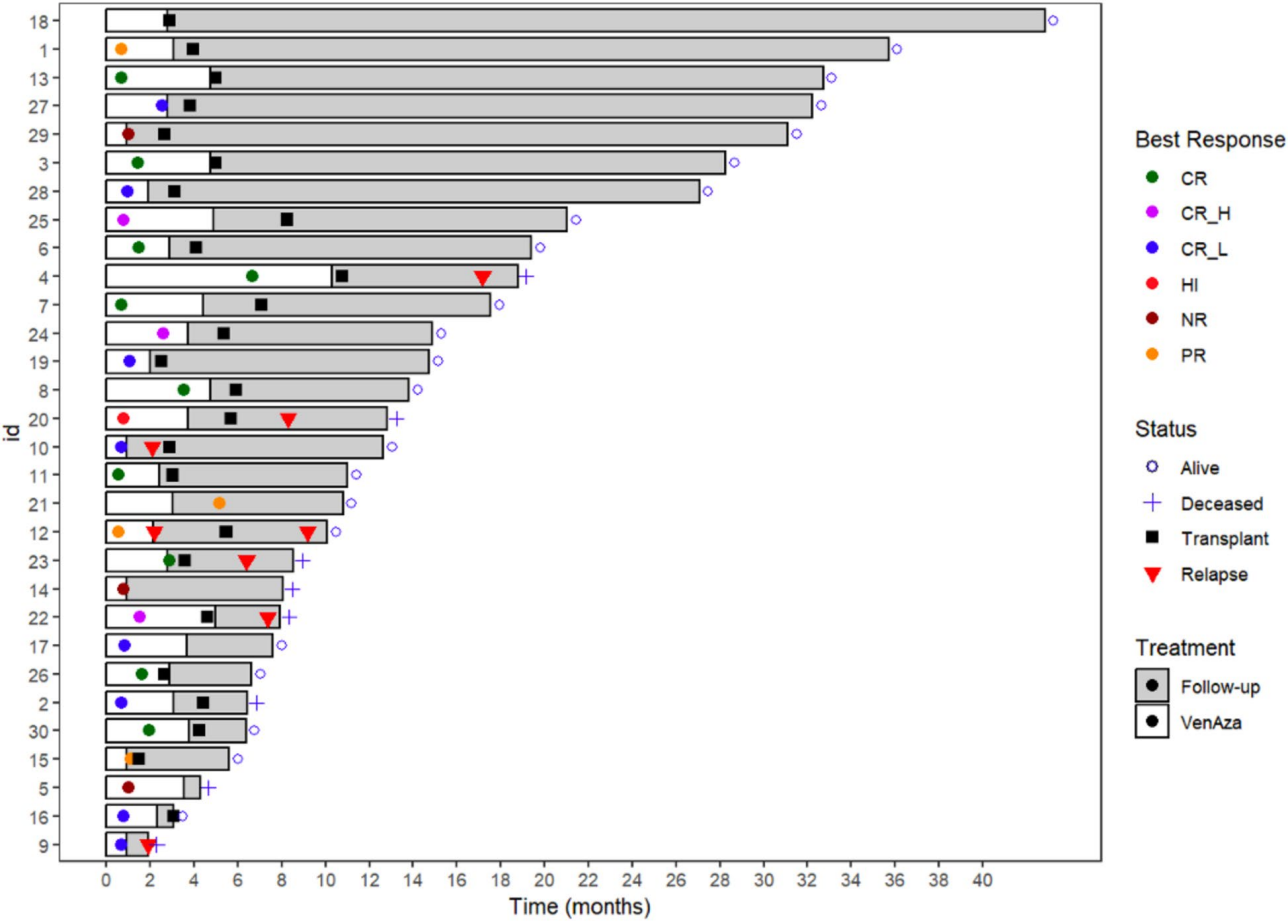
Swimmer plot of treatment duration, best response, allo-SCT and relapse for each patient. HMA/VEN was started at time 0. White areas indicate the duration HMA/VEN treatment. Grey areas indicate the duration of follow-up. Black squares indicate the time of allo-SCT. Colored dots indicate the time of best response. Red triangles indicate the time of relapse. The status of each patient (alive or deceased) at the time of data cutoff is shown at the end of each bar

Most responses were early, with the first and best responses achieved in cycle 1. Only two patients needed more than one cycle to reach their first response. The median time to best response was one cycle (range, 1–6). Twenty-two patients reached best response in cycle 1, while four required three cycles, three required two cycles, and one required six cycles to reach best response (Fig. 4).

According to IWG 2023 criteria, ORR was 83% (10/12) for the R/R patients and 94 (17/18) for the treatment-naïve patients (p = 0.55). CRc was 67% (8/12) and 88% (16/18), respectively (p = 0.08). There were no significant differences in ORR or CRc between HR-MDS vs CMML. Considering MDS patients, there were 8 CR (36%), five CRL (23%), four CRh (18%), two PR (10%), one HI (3%), and two no response (NR, 10%). According to Savona Criteria, among CMML patients (n = 7), three patients presented CR (43%), four presented MCR (57%), and one a PR (14%). We did not observed differences in response rate depending on *TP53* mutational status. Figure 3B shows details of overall response rates.

### Allo-SCT characteristics and results

Twenty-five patients (83%) underwent allo-SCT after HMA/VEN treatment, two of whom required a second bridging therapy before proceeding to allo-SCT. These two patients achieved CR and PR with HMA/VEN, respectively, but later progressed. They were rescued with CPX-351 and achieved a second CR and a CRh, respectively, and were able to undergo allo-SCT (Fig. 1). Overall transplant rate for patients bridged exclusively with HMA/VEN was 76.6% (23/30) for the entire cohort, 83% (15/18) for treatment-naïve patients, and 66.6% (8/12) for R/R patients.

Reasons for not proceeding to allo-SCT were varied. Three patients progressed and died. Two of the three were HR-MDS patients who had NR, and one was a CMML patient who attained a marrow CR (mCR) at the first cycle but later progressed and died. In addition, one HR-MDS patient did not proceed to allo-SCT because of the physician’s decision. This patient had relapsed after a prior allo-SCT and had started HMA/VEN with the intention of undergoing a second allo-SCT. The patient achieved a CR and later developed chronic GVHD, and it was decided not to carry out a second allo-SCT. Other CMML patient lost eligibility due to poor performance status after a long period of hospital admission for acute gastrointestinal bleeding due to a cytomegalovirus infection without G3/4 neutropenia. Both are currently in CR and mCR at the moment of writing this manuscript, respectively.

Allo-SCT was performed after a median of three cycles of HMA/VEN (range, 1–9). Five patients received only one cycle and four received four cycles (Fig. 4). Among the five patients that did not reach allo-SCT the median number of cycles received were 2 (range, 1–3). Transplant characteristics are described in Supplementary Table 1.

### Survival

**Fig. 5:**
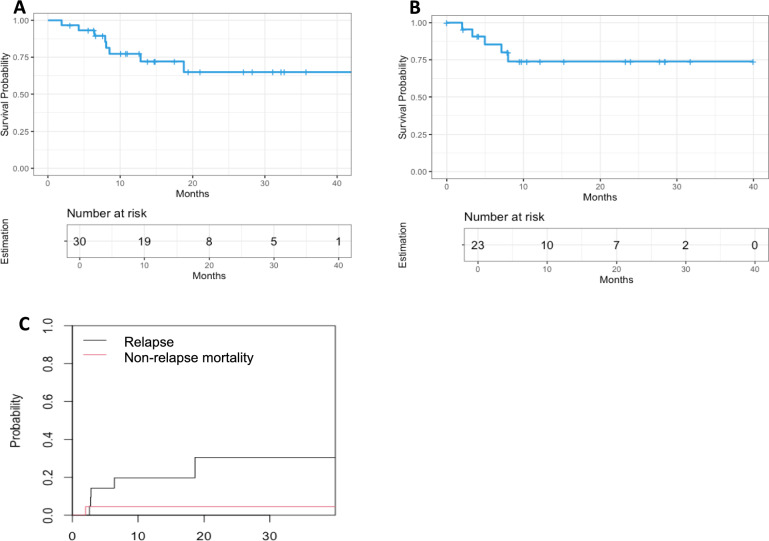
Overall survival (OS) and cumulative incidence of relapse (CIR). **A** OS of the entire cohort. **B** Post-allo-SCT OS of the 23 who underwent allo-SCT after HMA/VEN treatment. **C** Post-allo-SCT CIR (black line) and cumulative incidence of non-relapse mortality (red line)

With a median follow-up of 15.79 months, one-year and two-year OS of the entire cohort was 77.3% (95% CI ± 16%) and 64.9% (95% CI ± 21%), respectively (Fig. 5A). Two-year overall survival (OS) was 66% (95% CI ± 23) for HR-MDS patients and 73% (95% CI ± 32) for CMML patients (p = 0.53) (Supplementary Fig. 3A). For the 23 patients that underwent allo-SCT after HMA/VEN treatment, one-year and two-year post-allo-SCT OS was 75% (95% CI ± 18%) (Fig. 5B). Five patients relapsed after allo-SCT, resulting in a one-year CIR of 19.7% (95% CI ± 17.6%) and a two-year CIR of 30.5% (95% CI ± 25.4%). Two-year non-relapse mortality was 4.5%; only one patient died – from acute GVHD while in CR (Fig. 5C).

Of the 23 patients undergoing allo-SCT, only two suffered G3/4 acute GVHD and none had moderate or severe chronic GVHD. Median one-year and two-year GRFS were 74% (95% CI ± 19%) and 64% (95% CI ± 25%), respectively.

Two-year post-allo-SCT OS was 74.8% (95% CI ± 18.2) for HR-MDS patients and 83% (95% CI ± 29.8) for CMML patients (p = 0.97) (Supplementary Fig. 3B). Two-year post-allo-SCT OS was 76.6% (95% CI ± 23.7) for treatment-naïve patients and 71% (95% CI ± 33.5) for R/R patients (p = 0.91). Two-year post-allo-SCT OS was 50% (95% CI ± 21) for patients with TP53 mutations and 79% (95% CI ± 21) for those with wild-type TP53 (p = 0.19). Two-year post-allo-SCT CIR was 75% (95% CI ± 17) for those with TP53 mutations and 15.4% (95% CI ± 56) for those without mutations (p = 0.028) (Supplementary Fig. 1A and B). Only five patients did not reach allo-SCT in CRc. One-year post-allo-SCT OS was 72% (95% CI ± 24) for those in CRc and 80% (95% CI ± 35) for those not in CRc (p = 0.7) (Supplementary Fig. 1C).

### MRD pre- and post-allo-SCT

**Fig. 6:**
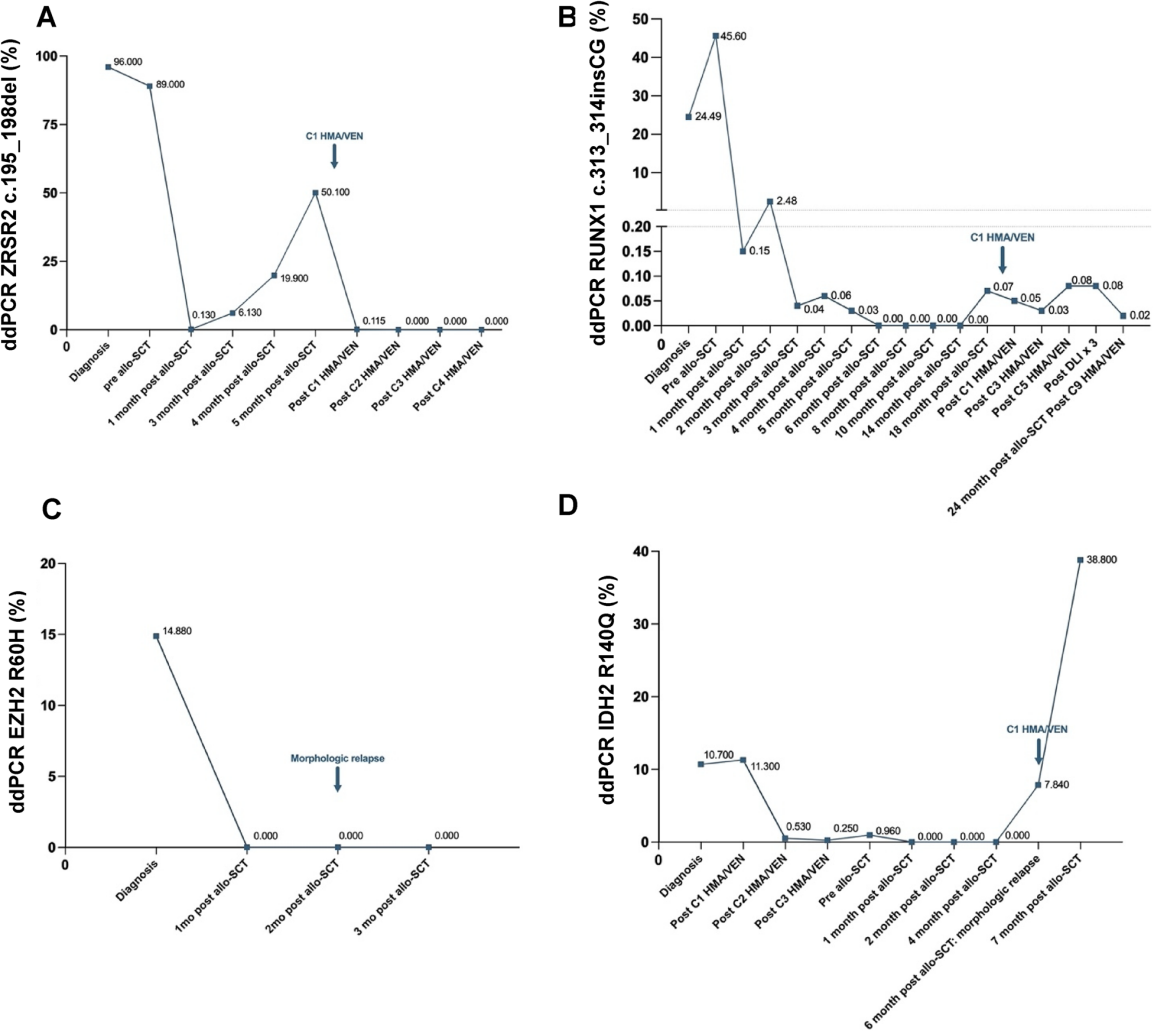
Molecular follow-up of mutations by droplet digital PCR (ddPCR) in four patients. **A** Monitoring of ZRSR2 c.195_198del alteration in sequential samples of a CMML patient detected early molecular relapse three months after allo-SCT. **B** An MDS-IB2 patient with RUNX1 c.313_314insCG molecular positivization nine months after allo-SCT. In sequential samples, this mutation was detected at a low level two years after allo-SCT but the patient remained in morphologic CR. **C** An EZH2 R690H mutation was present at diagnosis of an MDS-IB2 patient. The mutation was not detected in sequential samples but the patient suffered a morphological relapse two months after allo-SCT. **D** An IDH2 R140Q mutation was detected in an MDS-IB2 patient at the time of a morphological relapse six months post-allo-SCT

First, we evaluated the mutational profile by targeted NGS before allo-SCT in 17 patients (Fig. 6B). In 14 (82%), NGS detected a non-DTA mutation. Among these patients, there were six CR, three CR_L_, one CRh, one HI, and three PR. Positive pre-transplant MRD was not associated with worse post-allo-SCT OS. Two-year post-allo-SCT OS was 79% (95 CI ± 25) for patients without pre-transplant MRD and 67% (95 CI ± 52) for those with MRD (p = 0.49).

Besides conventional monitoring, we also performed personalized molecular monitoring with ddPCR as part of post-allo-SCT follow-up. Of the 14 patients who proceeded to allo-SCT in our center, ddPCR post-transplant monitoring was performed in nine, two of whom experienced an early molecular relapse detected by ddPCR. One patient had CMML-1 with ZRSR2 c.195_198del alteration at diagnosis. This patient experienced a molecular relapse three months after allo-SCT, which was confirmed two months later after immunosuppression tapering. He received preemptive treatment with DEC plus VEN and attained molecular negativization after cycle 2 that was sustained until cycle 5 (at data cutoff) (Fig. 6A). The second patient had MDS-IB2 and showed RUNX1 c.313_314insCG molecular positivization nine months after allo-SCT. He received preemptive treatment with HMA/VEN for nine cycles followed by donor lymphocyte infusion. At data cutoff two years after allo-SCT, he remained in morphologic CR despite a slight molecular RUNX1 c.313_314insCG mutation detectable by ddPCR (Fig. 6B). Two patients suffered a post-allo-SCT morphological relapse during molecular follow-up. One had MDS-IB2 and was monitored for the EZH2 R690H mutation quantification. He suffered an EZH2 R690H-negative morphological relapse two months after allo-SCT (Fig. 6C). The other patient also hadand was being monitored for IDH2 mutations. His morphological relapse occurred six months post-allo-SCT with no previous detection of IDH2 mutations by ddPCR. The IDH2 mutation was detected at the time of the morphological relapse. The patient was treated with HMA/VEN but did not respond (Fig. 6D).

In summary, four patients received post allo-SCT disease-modifying treatments. As previously mentioned, two patients were treated with preemptive HMA/VEN following molecular positivization and remain in a CR. And two additional patients received HMA/VEN after a morphological relapse without response.

Targeted NGS was also performed at relapse in nine patients. One relapsed prior to allo-SCT with acquisition of an SH2B3 mutation (c.1315delC). Of the eight patients who relapsed post-allo-SCT, one acquired an NRAS mutation, another lost an IDH2 and a STAG2 mutation, a third lost a SH2B3 and a ZRSR2 mutation, and the remaining five maintained their baseline mutational profile (Fig. 6C).

## Discussion

In the present real-world study, we have retrospectively analyzed 30 patients who were deemed eligible for allo-SCT and who were treated with HMA/VEN as a bridge to allo-SCT. We observed a promising ORR (90%), transplant rate (76.6%), and OS (two-year post-allo-SCT OS 75%).

The ORR in our patients (90%) was higher than that usually attained with HMA in monotherapy (< 50%) [14], even tough given the retrospective design and the limitations in comparing across studies, this conclusion should be interpreted with caution. Moreover, responses were achieved early, mostly during the first cycle. This early response rate is in line with previous reports in HR-MDS [20, 45] and CMML [29–31], where HMA/VEN was used mostly with non-bridging objectives. Early response is particularly important for a bridging therapy, as it can reduce the number of dropouts since patients can undergo allo-SCT before disease progression. Furthermore, early response can limit the risk of treatment-related toxicity and cytopenia.

An essential concern in any bridging strategy is the safety profile. Thrombocytopenia or neutropenia occurred in 70% and 85%, respectively, of our patients, most cases occurred during the first 30 days of treatment. Seventeen per cent of patients experienced febrile neutropenia requiring hospital admission during the first cycle. However, none of our patients died in the first 30 days, and only one patient died—without treatment-related toxicity—due to disease progression in the first 60 days. Moreover, only one of our R/R patients lost eligibility for allo-SCT due to poor performance status after a long period of hospital admission. This patient had CMML-1 and suffered iatrogenic acute gastrointestinal bleeding without G3/4 cytopenia. At the time of data cutoff, she was still in mCR. Moreover, two patients who required a second bridging therapy with intensive chemotherapy (CPX-531) after HMA/VEN were able to tolerate it and later proceeded to allo-SCT. Our findings of a good safety profile are in line with previous studies reporting manageable toxicities [27, 30, 32, 33, 46, 47].

An important issue related to safety and tolerability is patient age and frailty. Six of our patients were older than 70 years. Four reached allo-SCT, one progressed before allo-SCT, and the other one did not proceed to allo-SCT because of the physician’s decision. Of the four elderly patients who underwent allo-SCT, three were alive and in CR at data cutoff and one had died due to disease progression. Other studies have also found that older fit patients can benefit from allo-SCT [48, 49]. We therefore conclude that many elderly patients may benefit from HMA/VEN followed by allo-SCT. However, we suggest assessing eligibility on an individual basis—rather than only on patient age—and closely monitoring elderly patients during treatment.

Twenty-five patients out of thirty (83%) underwent allo-SCT. The transplant rate after HMA/VEN in our study was 76.6%, higher than the ratio reported previously with HMA as monotherapy [4, 14, 50, 51]. However, due to the inherent limitations in cross-study comparisons, this conclusion should be interpreted with caution and needs to be validated in larger, prospective studies.

Some preliminary studies with small patient cohorts included some patients who were transplanted after receiving HMA/VEN [20, 27–30, 32, 33]. Recently, an international phase 1b study including 107 first-line HR-MDS reported that 43% of the cohort proceeded to SCT, which is higher than other reported SCT rates, with a reasonable safety profile [9]. However, none of these studies included specifically and exclusively patients who received HMA/VEN as a cytoreductive therapy with the aim of bridging to allo-SCT. To the best of our knowledge, this is the first study focusing on patients eligible for allo-SCT and reporting a promising transplant rate after HMA/VEN. Our findings will be useful in daily clinical practice when deciding whether a patient will be able to undergo allo-SCT, the only potentially curative treatment available.

Our findings on OS were encouraging, with one-year and two-year OS of 77.3% and 64.9%, respectively. The VidazaAllo study reported a 3-year OS for the azacytidine followed by allo-SCT arm of 50%. However, due to the relatively short follow-up of our study, direct comparison may not be adequate. Moreover, the OS benefit can only be addressed in context of a randomized clinical trial. For the 23 patients who proceeded to allo-SCT directly after HMA/VEN treatment, one-year and two-year post-allo-SCT OS was also promising (75%), which is along the lines of previous reports [27, 28, 33]. Again, there were no differences between HR-MDS and CMML patients or between treatment-naïve and R/R patients, though this may be due to our relatively small sample size. Larger cohorts are needed to properly assess differences between groups.

R/R patients with HR-MDS or CMML have dismal outcomes, with OS shorter than six months [21, 52]. Our study included 12 R/R patients, most of whom had previously been treated with HMA. The outcome in these patients is encouraging: their ORR was 83%, which is in line with other studies of HMA/VEN [21, 25–27], and their OS and post-allo-SCT OS were similar to those of our treatment-naïve patients. Moreover, 66.6% of our R/R patients effectively bridged to allo-SCT after HMA/VEN.

Of the seven CMML and one aCML patients in our study, one (with myeloproliferative disease) progressed before allo-SCT and another attained CR at the moment of data cut-off but lost eligibility for allo-SCT due to poor performance status after a prolonged hospital admission. The remaining six, including the aCML patient, proceeded to allo-SCT and none relapsed. Interestingly, four of our seven CMML patients had myeloproliferative disease. All four responded to HMA/VEN and three underwent allo-SCT. Previous studies had reported that HMA/VEN could lead to a high response rate in CMML patients [29–31], but these responses appeared to be brief, with early relapses, leading the investigators to question the role of this therapy in CMML. Nevertheless, in the context of HMA/VEN as a bridging therapy to transplant, long-lasting responses may not be needed, which suggests a strong therapeutic potential for this combination.

Findings are inconsistent on whether pre-allo-SCT response correlates with post-allo-SCT outcome [53]. In our study, only five patients had not attained CRc at the time of allo-SCT, and their post-allo-SCT OS was not different from the patients that underwent allo-SCT while in CR. In addition, our review of pre-allo-SCT NGS findings on 17 transplanted patients showed that only three patients reached allo-SCT with no mutations detected, while 14 showed at least one (non-DTA) mutation. In AML, flow-detected negative MRD after VEN/AZA has been reported to be 40%, in 50% of the patients reached after the fourth treatment cycle [4]. Post-allo-SCT OS of our patients with NGS-detected negative MRD before transplant was similar to those with mutations. This analysis had some limitations, including the small number of patients included and the lack of error-corrected NGS. Therefore, future studies with larger cohorts and longer follow-ups are warranted to clarify this matter.

Our two-year CIR was 30.5%, with five patients who relapsed after allo-SCT. Ours is one of the first studies evaluating MDS/CMML post-allo-SCT CIR after bridging with HMA/VEN, and the CIR is not higher than that reported for patients bridged with HMA as monotherapy [10, 53, 54]. Patients with TP53 mutations had a higher two-year CIR than those without TP53 mutations (75% vs 15.4%; p = 0.028). Most of the relapses occurred during the first six months post-transplant. However, only six of our patients harbored TP53 mutations, indicating a need for further investigation with larger cohorts.

The role of molecular follow-up is critical to identify molecular relapse and implement preemptive treatment. In addition to conventional monitoring after allo-SCT, we are progressively implementing personalized molecular follow-up with ddPCR in our center. We had ddPCR results available on nine patients who underwent allo-SCT. ddPCR detected a molecular relapse in two of these patients, and they were able to start preemptive treatment with HMA/VEN with good outcomes. Despite the small number of patients with ddPCR results available, the case of these two patients highlights the need for molecular follow-up on patients after transplant.

Our study has several limitations. First, this is a retrospective work. Second, it includes a relatively small number of patients that limits the generalizability of the findings. In addition, follow-up—though similar to that of previous studies [23, 24]—was relatively short. Given this short follow-up period and the retrospective nature of this study, we are unable to determine whether there are differences in terms of patient survival, and results should be carefully validated by future larger randomized prospective studies.

Nevertheless, to the best of our knowledge, this is the first study including exclusively HR-MDS and CMML patients who were eligible for allo-SCT. As such, our findings of good transplant and OS rates together with a manageable toxicity profile provide preliminary yet solid evidence for the beneficial impact of HMA/VEN as a bridging strategy to allo-SCT. Our real-world results warrant validation in larger prospective cohorts and may well help to increase the number of HR-MDS and CMML patients who are able to undergo allo-SCT.

## Supplementary Information

Additional file1 (DOCX 797 KB)

## Data Availability

The datasets generated during and/or analyzed during the current study are available from the corresponding author on reasonable request.

## Abbreviations

HR-MDS: High-risk myelodysplastic syndromes
CMML: Chronic myelomonocytic leukemia
MDS/MPN: Myelodysplastic syndromes/myeloproliferative neoplasms
allo-SCT: Allogeneic stem cell transplantation
HMA: Hypomethylating agents
HMA/VEN: HMA with venetoclax
ORR: Overall response rate
GRFS: GVHD-free/relapse-free survival
MRD: Minimal residual disease
BCL-2: B-cell lymphoma 2 protein

## References

[1] Nakamura R, Saber W, Martens MJ, Ramirez A, Scott B, Oran B, et al. Biologic assignment trial of reduced-intensity hematopoietic cell transplantation based on donor availability in patients 50–75 years of age with advanced myelodysplastic syndrome. J Clin Oncol. 2021;39(30):3328–39. 34106753 10.1200/JCO.20.03380 PMC8791814

[2] Pfeilstocker M, Tuechler H, Sanz G, Schanz J, Garcia-Manero G, Sole F, et al. Time-dependent changes in mortality and transformation risk in MDS. Blood. 2016;128(7):902–10. 27335276 10.1182/blood-2016-02-700054 PMC5161006

[3] Bernard E, Tuechler H, Greenberg PL, Hasserjian RP, Arango Ossa JE, Nannya Y, et al. Molecular international prognostic scoring system for myelodysplastic syndromes. NEJM Evid. 2022;1(7):EVIDoa200008. 10.1056/EVIDoa2200008 38319256

[4] Kroger N, Sockel K, Wolschke C, Bethge W, Schlenk RF, Wolf D, et al. Comparison between 5-azacytidine treatment and allogeneic stem-cell transplantation in elderly patients with advanced mds according to donor availability (VidazaAllo Study). J Clin Oncol. 2021;39(30):3318–27. 34283629 10.1200/JCO.20.02724

[5] Versluis J, Saber W, Tsai HK, Gibson CJ, Dillon LW, Mishra A, et al. Allogeneic hematopoietic cell transplantation improves outcome in myelodysplastic syndrome across high-risk genetic subgroups: genetic analysis of the blood and marrow transplant clinical trials network 1102 study. J Clin Oncol. 2023;41(28):4497–510. 37607457 10.1200/JCO.23.00866 PMC10552956

[6] Garcia JS, Kim HT, Murdock HM, Ansuinelli M, Brock J, Cutler CS, et al. Prophylactic maintenance with venetoclax/azacitidine after reduced-intensity conditioning allogeneic transplant for high-risk MDS and AML. Blood Adv. 2024;8(4):978–90. 38197938 10.1182/bloodadvances.2023012120 PMC10883823

[7] Platzbecker U, Wermke M, Radke J, Oelschlaegel U, Seltmann F, Kiani A, et al. Azacitidine for treatment of imminent relapse in MDS or AML patients after allogeneic HSCT: results of the RELAZA trial. Leukemia. 2012;26(3):381–9. 21886171 10.1038/leu.2011.234 PMC3306138

[8] Atallah E, Logan B, Chen M, Cutler C, Deeg J, Jacoby M, et al. Comparison of patient age groups in transplantation for myelodysplastic syndrome: the medicare coverage with evidence development study. JAMA Oncol. 2020;6(4):486–93. 31830234 10.1001/jamaoncol.2019.5140 PMC6990739

[9] Garcia JS, Platzbecker U, Odenike O, Fleming SA, Yew Fong C, et al. Efficacy and safety of venetoclax plus azacitidine for patients with treatment-naive high-risk myelodysplastic syndromes. Blood. 2024;145(11):1126–35. 10.1182/blood.2024025464 PMC11923426 39652823

[10] de Witte T, Bowen D, Robin M, Malcovati L, Niederwieser D, Yakoub-Agha I, et al. Allogeneic hematopoietic stem cell transplantation for MDS and CMML: recommendations from an international expert panel. Blood. 2017;129(13):1753–62. 28096091 10.1182/blood-2016-06-724500 PMC5524528

[11] Fenaux P, Mufti GJ, Hellstrom-Lindberg E, Santini V, Finelli C, Giagounidis A, et al. Efficacy of azacitidine compared with that of conventional care regimens in the treatment of higher-risk myelodysplastic syndromes: a randomised, open-label, phase III study. Lancet Oncol. 2009;10(3):223–32. 19230772 10.1016/S1470-2045(09)70003-8 PMC4086808

[12] Garcia-Manero G, Jabbour E, Borthakur G, Faderl S, Estrov Z, Yang H, et al. Randomized open-label phase II study of decitabine in patients with low- or intermediate-risk myelodysplastic syndromes. J Clin Oncol. 2013;31(20):2548–53. 23733767 10.1200/JCO.2012.44.6823 PMC4878053

[13] Kantarjian H, Issa JP, Rosenfeld CS, Bennett JM, Albitar M, DiPersio J, et al. Decitabine improves patient outcomes in myelodysplastic syndromes: results of a phase III randomized study. Cancer. 2006;106(8):1794–803. 16532500 10.1002/cncr.21792

[14] Saygin C, Carraway HE. Current and emerging strategies for management of myelodysplastic syndromes. Blood Rev. 2021;48:100791. 33423844 10.1016/j.blre.2020.100791

[15] Baranwal A, Chhetri R, Yeung D, Clark M, Shah S, Litzow MR, et al. Factors predicting survival following alloSCT in patients with therapy-related AML and MDS: a multicenter study. Bone Marrow Transplant. 2023;58(7):769–76. 37012415 10.1038/s41409-023-01970-0

[16] DeFilipp Z, Ciurea SO, Cutler C, Robin M, Warlick ED, Nakamura R, et al. Hematopoietic cell transplantation in the management of myelodysplastic syndrome: an evidence-based review from the American society for transplantation and cellular therapy committee on practice guidelines. Transplant Cell Ther. 2023;29(2):71–81. 36436780 10.1016/j.jtct.2022.11.014

[17] Adams CM, Clark-Garvey S, Porcu P, Eischen CM. Targeting the Bcl-2 family in B cell lymphoma. Front Oncol. 2018;8:636. 30671383 10.3389/fonc.2018.00636 PMC6331425

[18] Ganan-Gomez I, Yang H, Ma F, Montalban-Bravo G, Thongon N, Marchica V, et al. Stem cell architecture drives myelodysplastic syndrome progression and predicts response to venetoclax-based therapy. Nat Med. 2022;28(3):557–67. 35241842 10.1038/s41591-022-01696-4 PMC8938266

[19] DiNardo CD, Jonas BA, Pullarkat V, Thirman MJ, Garcia JS, Wei AH, et al. Azacitidine and venetoclax in previously untreated acute myeloid leukemia. N Engl J Med. 2020;383(7):617–29. 32786187 10.1056/NEJMoa2012971

[20] Garcia JS, Wei AH, Borate U, Fong CY, Baer MR, Nolte F, et al. Safety, efficacy, and patient-reported outcomes of venetoclax in combination with azacitidine for the treatment of patients with higher-risk myelodysplastic syndrome: a phase 1b study. Blood. 2020;136:55–7.

[21] Zeidan AM, Borate U, Pollyea DA, Brunner AM, Roncolato F, Garcia JS, et al. A phase 1b study of venetoclax and azacitidine combination in patients with relapsed or refractory myelodysplastic syndromes. Am J Hematol. 2023;98(2):272–81. 36309981 10.1002/ajh.26771 PMC10100228

[22] Khanam R, Shahzad M, Chaudhary SG, Ali F, Shah Z, Pachika PS, et al. Outcomes after venetoclax with hypomethylating agents in myelodysplastic syndromes: a systematic review and meta-analysis. Leuk Lymphoma. 2022;63(11):2671–8. 35687838 10.1080/10428194.2022.2084730

[23] Levitz D, Saunthararajah Y, Fedorov K, Shapiro LC, Mantzaris I, Shastri A, et al. A metabolically optimized, noncytotoxic low-dose weekly decitabine/venetoclax in MDS and AML. Clin Cancer Res. 2023;29(15):2774–80. 37341641 10.1158/1078-0432.CCR-23-0842

[24] Masetti R, Baccelli F, Leardini D, Gottardi F, Vendemini F, di Gangi A, et al. Venetoclax-based therapies in pediatric advanced MDS and relapsed/refractory AML: a multicenter retrospective analysis. Blood Adv. 2023;7(16):4366–70. 37216275 10.1182/bloodadvances.2023010113 PMC10432591

[25] Bazinet A, Darbaniyan F, Jabbour E, Montalban-Bravo G, Ohanian M, et al. Azacitidine plus venetoclax in patients with high-risk myelodysplastic syndromes or chronic myelomonocytic leukaemia: phase 1 results of a single-centre, dose-escalation, dose-expansion, phase 1–2 study. Lancet Haematol. 2022. 10.1016/S2352-3026(22)00216-2. 36063832 10.1016/S2352-3026(22)00216-2

[26] Prebet T, Gore SD, Esterni B, Gardin C, Itzykson R, Thepot S, et al. Outcome of high-risk myelodysplastic syndrome after azacitidine treatment failure. J Clin Oncol. 2011;29(24):3322–7. 21788559 10.1200/JCO.2011.35.8135 PMC4859209

[27] Ball BJ, Famulare CA, Stein EM, Tallman MS, Derkach A, Roshal M, et al. Venetoclax and hypomethylating agents (HMAs) induce high response rates in MDS, including patients after HMA therapy failure. Blood Adv. 2020;4(13):2866–70. 32589727 10.1182/bloodadvances.2020001482 PMC7362378

[28] Bataller A, Montalban-Bravo G, Bazinet A, Alvarado Y, Chien K, Venugopal S, et al. Oral decitabine plus cedazuridine and venetoclax in patients with higher-risk myelodysplastic syndromes or chronic myelomonocytic leukaemia: a single-centre, phase 1/2 study. Lancet Haematol. 2024;11(3):e186–95. 38316133 10.1016/S2352-3026(23)00367-8

[29] Ball S, Jain AG, Aguirre LE, Al Ali N, Zhang Y, Chan O, et al. Hypomethylating agent and venetoclax in patients with chronic myelomonocytic leukemia: is the combination indeed better? Am J Hematol. 2022;97(5):E185–8. 35179241 10.1002/ajh.26504

[30] Montalban-Bravo G, Hammond D, DiNardo CD, Konopleva M, Borthakur G, Short NJ, et al. Activity of venetoclax-based therapy in chronic myelomonocytic leukemia. Leukemia. 2021;35(5):1494–9. 33846541 10.1038/s41375-021-01240-2

[31] Saliba AN, Litzow MR, Gangat N, Al-Kali A, Foran JM, Hogan WJ, et al. Outcomes of venetoclax-based therapy in chronic phase and blast transformed chronic myelomonocytic leukemia. Am J Hematol. 2021;96(11):E433–6. 34428328 10.1002/ajh.26334

[32] Komrokji RS, Singh AM, Ali NA, Chan O, Padron E, Sweet K, et al. Assessing the role of venetoclax in combination with hypomethylating agents in higher risk myelodysplastic syndrome. Blood Cancer J. 2022;12(11):148. 36329025 10.1038/s41408-022-00744-z PMC9633639

[33] Gangat N, McCullough K, Johnson I, Al-Kali A, Begna KH, Patnaik MM, et al. Real-world experience with venetoclax and hypomethylating agents in myelodysplastic syndromes with excess blasts. Am J Hematol. 2022;97(6):E214–6. 35303376 10.1002/ajh.26539

[34] Mei C, Ye L, Ren Y, Zhou X, Ma L, Xu G, et al. 15-days duration of venetoclax combined with azacitidine in the treatment of relapsed/refractory high-risk myelodysplastic syndromes: a retrospective single-center study. Hematol Oncol. 2023;41(3):546–54. 36516239 10.1002/hon.3112

[35] Yang TT, Song XL, Zhao YM, Ye BD, Luo Y, Xiao HW, et al. Outcome after allogeneic hematopoietic stem cell transplantation following Venetoclax-based therapy among AML and MDS patients. Ann Hematol. 2022;101(12):2731–41. 36318288 10.1007/s00277-022-04983-9

[36] Zeidan AM, Platzbecker U, Bewersdorf JP, Stahl M, Ades L, Borate U, et al. Consensus proposal for revised international working group 2023 response criteria for higher-risk myelodysplastic syndromes. Blood. 2023;141(17):2047–61. 36724453 10.1182/blood.2022018604

[37] Savona MR, Malcovati L, Komrokji R, Tiu RV, Mughal TI, Orazi A, et al. An international consortium proposal of uniform response criteria for myelodysplastic/myeloproliferative neoplasms (MDS/MPN) in adults. Blood. 2015;125(12):1857–65. 25624319 10.1182/blood-2014-10-607341 PMC4915792

[38] Cheson BD, Greenberg PL, Bennett JM, Lowenberg B, Wijermans PW, Nimer SD, et al. Clinical application and proposal for modification of the International Working Group (IWG) response criteria in myelodysplasia. Blood. 2006;108(2):419–25. 16609072 10.1182/blood-2005-10-4149

[39] Dohner H, Wei AH, Appelbaum FR, Craddock C, DiNardo CD, Dombret H, et al. Diagnosis and management of AML in adults: 2022 recommendations from an international expert panel on behalf of the ELN. Blood. 2022;140(12):1345–77. 35797463 10.1182/blood.2022016867

[40] McGowan-Jordan J, Hastings R, Moore S. Re: international system for human cytogenetic or cytogenomic nomenclature (ISCN): some thoughts, by T. Liehr. Cytogenet Genome Res. 2021;161(5):225–6. 34407535 10.1159/000516655

[41] Gorello P, Cazzaniga G, Alberti F, Dell’Oro MG, Gottardi E, Specchia G, et al. Quantitative assessment of minimal residual disease in acute myeloid leukemia carrying nucleophosmin (NPM1) gene mutations. Leukemia. 2006;20(6):1103–8. 16541144 10.1038/sj.leu.2404149

[42] Snowden JA, Sanchez-Ortega I, Corbacioglu S, Basak GW, Chabannon C, de la Camara R, et al. Indications for haematopoietic cell transplantation for haematological diseases, solid tumours and immune disorders: current practice in Europe, 2022. Bone Marrow Transplant. 2022;57(8):1217–39. 35589997 10.1038/s41409-022-01691-w PMC9119216

[43] Jagasia MH, Greinix HT, Arora M, Williams KM, Wolff D, Cowen EW, et al. National institutes of health consensus development project on criteria for clinical trials in chronic graft-versus-host disease: I. the 2014 diagnosis and staging working group report. Biol Blood Marrow Transplant. 2015;21(3):389–401. 25529383 10.1016/j.bbmt.2014.12.001 PMC4329079

[44] Harris AC, Young R, Devine S, Hogan WJ, Ayuk F, Bunworasate U, et al. International, multicenter standardization of acute graft-versus-host disease clinical data collection: a report from the Mount Sinai acute GVHD international consortium. Biol Blood Marrow Transplant. 2016;22(1):4–10. 26386318 10.1016/j.bbmt.2015.09.001 PMC4706482

[45] Garcia JS, Wei AH, Jacoby MA, Fong CY, Borate U, Baer MR, et al. Molecular responses are observed across mutational spectrum in treatment-naïve higher-risk myelodysplastic syndrome patients treated with venetoclax plus azacitidine. Blood. 2021. 10.1182/blood-2021-145613.33181835

[46] Garcia JS, Platzbecker U, Odenike O, Fleming S, Fong CY, Cook R, et al. Efficacy and safety of venetoclax in combination with azacitidine for the treatment of patients with treatment-naive higher-risk myelodysplastic syndromes. Blood. 2023. 10.1182/blood-2023-189446.37053555

[47] Garcia-Manero G, Odenike O, Fleming S, Roboz GJ, Jacoby M, Cunningham I, et al. Combination of venetoclax and azacitidine in patients with treatment-naive, high-risk myelodysplastic syndromes with responses leading to stem cell transplantation. Blood. 2023. 10.1182/blood-2023-187102.37441846

[48] Maffini E, Ngoya M, Galimard JE, Harbi S, Kroger N, Platzbecker U, et al. Allogeneic hematopoietic cell transplantation for patients with AML aged 70 years or older in first remission. A study from the Acute Leukemia working party of the European society for blood and marrow transplantation (EBMT). Bone Marrow Transplant. 2023;58(9):1033–41. 37386253 10.1038/s41409-023-02027-y

[49] Heini AD, Berger MD, Seipel K, Taleghani BM, Baerlocher GM, Leibundgut K, et al. Consolidation with autologous stem cell transplantation in first remission is safe and effective in AML patients above 65 years. Leuk Res. 2017;53:28–34. 27978458 10.1016/j.leukres.2016.12.001

[50] Voso MT, Leone G, Piciocchi A, Fianchi L, Santarone S, Candoni A, et al. Feasibility of allogeneic stem-cell transplantation after azacitidine bridge in higher-risk myelodysplastic syndromes and low blast count acute myeloid leukemia: results of the BMT-AZA prospective study. Ann Oncol. 2017;28(7):1547–53. 28368509 10.1093/annonc/mdx154

[51] Getta BM, Kishtagari A, Hilden P, Tallman MS, Maloy M, Gonzales P, et al. Allogeneic hematopoietic stem cell transplantation is underutilized in older patients with myelodysplastic syndromes. Biol Blood Marrow Transplant. 2017;23(7):1078–86. 28336325 10.1016/j.bbmt.2017.03.020 PMC7386429

[52] Jabbour E, Garcia-Manero G, Batty N, Shan J, O’Brien S, Cortes J, et al. Outcome of patients with myelodysplastic syndrome after failure of decitabine therapy. Cancer. 2010;116(16):3830–4. 20564137 10.1002/cncr.25247 PMC4295788

[53] Garcia-Manero G. Myelodysplastic syndromes: 2023 update on diagnosis, risk-stratification, and management. Am J Hematol. 2023;98(8):1307–25. 37288607 10.1002/ajh.26984 PMC12002404

[54] Fenaux P, Platzbecker U, Ades L. How we manage adults with myelodysplastic syndrome. Br J Haematol. 2020;189(6):1016–27. 31568568 10.1111/bjh.16206

