# Supplementary material for: Hypomethylating agents plus venetoclax for high-risk MDS and CMML as bridge therapy to transplant: a GESMD study
Source: Exp Hematol Oncol. 2025 Apr 26;14:61. doi: 10.1186/s40164-025-00652-5 (PMC12032758; doi:10.1186/s40164-025-00652-5)
Supplement: Supplementary file 1 — Additional file1 (DOCX 797 KB) [file 40164_2025_652_MOESM1_ESM.docx]

**Supplementary Information**

**Hypomethylating agents plus venetoclax for high-risk MDS and CMML as bridge therapy to transplant: a GESMD study**

Ines Zugasti, Monica Lopez-Guerra, Sandra Castaño-Díez, Daniel Esteban, Alejandro Avendaño, Helena Pomares, Ana Perez, Sara García-Ávila, Irene Padilla Conejo, Cristina de la Fuente Montes, Alexandra Martínez-Roca, Beatriz Merchán, Carlos Jiménez-Vicente, Francisca Guijarro, Jose Ramón Álamo, Albert Cortes-Bullich, Victor Torrecillas, Lucia Mont, Esther Carcelero, Gisela Riu, Lurdes Zamora, Juan Bargay, Ana Triguero, Maria Suarez-Lledó, Maria Queralt Salas, Felix López-Cadenas, Fernando Ramos, Blanca Xicoy, David Valcárcel, Montserrat Arnan, Carmen Martínez, Montserrat Rovira, Francesc Fernández-Avilés, Maria Díez-Campelo, Jordi Esteve*, Marina Díaz-Beyá

**Supplementary Table 1. Allo-SCT characteristics in the 25 patients undergoing allo-SCT**

| **Characteristic** |  |
| --- | --- |
| ***Donor type*** | **n=25; n (%)** |
| Family donor | 10 (40) |
| HLA-identical family donor | 8 (80) |
| Haploidentical family donor | 2 (20) |
| Unrelated donor | 15 (60) |
| HLA-identical (8/8) | 11 (73) |
| HLA-mismatched (7/8) | 4 (27) |
| ***Conditioning type*** | **n=25; n (%)** |
| Myeloablative | 7 (28) |
| Reduced intensity | 18 (72) |
| ***Prophylaxis of Graft-versus-Host Disease (GVHD)*** | **n=24; n (%)** |
| Cyclophosphamide + Tacrolimus | 10 (42) |
| Cyclophosphamide + Tacrolimus + Mycophenolate | 1 (4.2) |
| Cyclophosphamide + Tacrolimus + Sirolimus | 7 (30) |
| Tacrolimus + Methotrexate | 1 (4.2) |
| Tacrolimus + Mycophenolate | 1 (4.2) |
| Tacrolimus + Sirolimus | 1 (4.2) |
| T-cell depletion *ex vivo* | 3 (13) |

**Supplementary Table 2**. Detailed Safety Characteristics.

| ***Safety Characteristics*** | ***n=30*** |
| --- | --- |
| Grade 3/4 neutropenia, n (%)  *Detailed for cycle 1:*  First line patients (*n* patients, %; median days)  R/R patients (*n* patients, %; median days)  MDS patients (*n* patients; median days)  CMML patients (*n* patients; median days) | 23 (85)  15 (83);20 days  8 (66); 22 days  17 (94); 17 days  6 (75); 22 days |
| Grade 3/4 thrombocytopenia n (%)  *Detailed for cycle 1:*  First line patients (*n* patients, %; median days)  R/R patients (*n* patients, %; median days)  MDS patients (*n* patients, %; median days)  CMML patients (*n* patients, %; median days) | 19 (70)  12 (63); 20 days  7 (58); 22 days  15 (83); 20 days  4 (50); 23 days |
| Grade 3/4 anemia n (%)  *Detailed for cycle 1:*  First line patients (n patients, %; median days)  R/R patients (n patients, %; median days)  MDS patients (n patients, %; median days)  CMML patients (n patients, %; median days) | 17(63)  9 (50); 18 days  8 (67); 16 days  13 (72); 12 days  4 (50); 20 days |
| **Patients that received a total of 1 or 2 cycles**  *Reason for treatment discontinuation*  Progression  Underwent allo-SCT | **n = 16 (64%)**  3  13 |
| ***VEN dose reduction requirements, n (%)***  ***Second cycle***  *1 SMD-EB1 (14 🡪 7 days)*  *1 LMCa (21 🡪 14 days)*  ***Third cycle:***  *1 SMD-EB1 (14 🡪7 days)*  *1 SMD-EB1 (28 🡪 7 days)*  ***Fourth cycle:***  *1 patient (28 🡪 14 days)* | **n = 30**  2/25 (8%)  2/15 (13%)  1/6 (16%) |

**Supplementary Figure 1. Post-allo-SCT overall survival (OS) and cumulative incidence of relapse (CIR) in 23 patients who proceeded directly to allo-SCT after HMA/VEN treatment.** **(A)** OS of TP53-mutated patients (red line) and TP53-wild-type patients (blue line). **(B)** Post-allo-SCT CIR of TP53-mutated patients (broken line) and TP53-wild-type patients (solid line). **(C)** Post-allo-SCT OS of the five patients who underwent allo-SCT while not in CRc (red line) and the 17 patients who underwent allo-SCT in CRc (blue line).


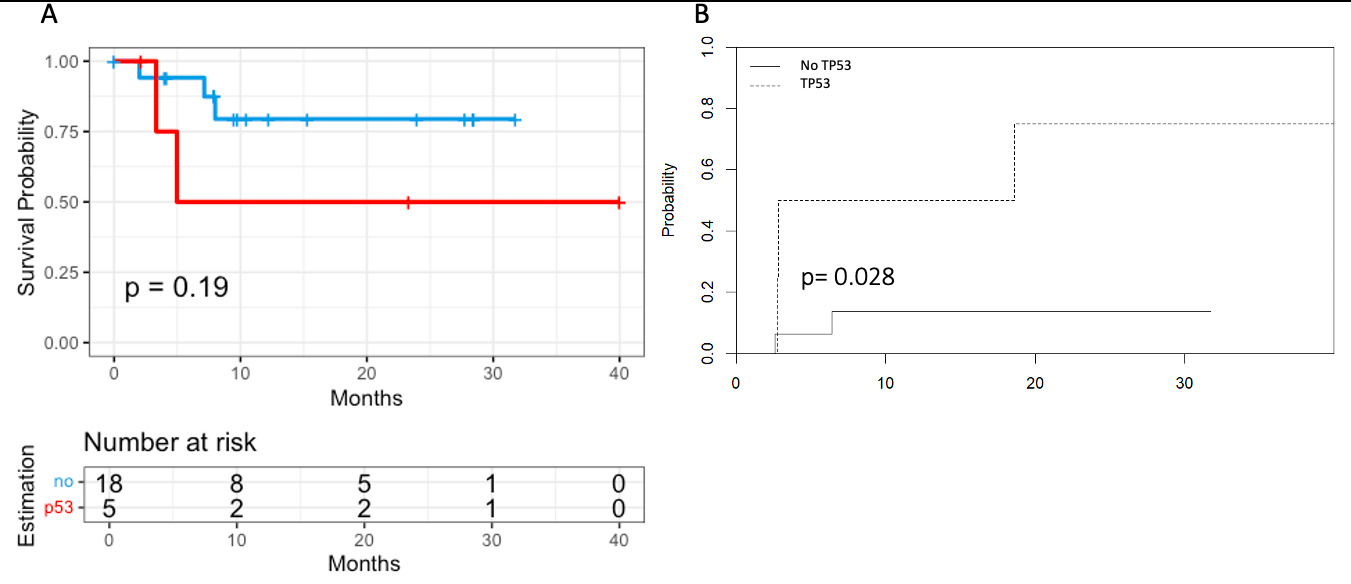

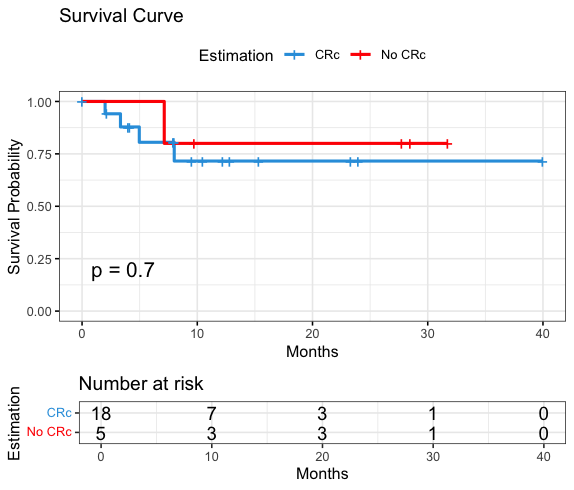


**A**

**B**

**C**

**Supplementary Figure 2.** Comparison of post allo-SCT overall survival of MDS/CMML patients who underwent allo-SCT at our institution depending of pre allo-SCT treatment (HMA/VEN in red, N= 23, Other bridging therapies in blue, N=68).

**Supplementary Figure 3.** Comparison of overall survival between MDS and CMML. Two-year overall survival (OS) was 66% (95% CI ± 23) for HR-MDS patients and 73% (95% CI ± 32) for CMML patients (p = 0.53) **(A).** Two-year OS post-allo-SCT was 74.8% (95% CI ± 18.2) for HR-MDS patients and 83% (95% CI ± 29.8) for CMML patients (p = 0.97) **(B).**

**A**

**B**
